# Differences in the Susceptibility to Commercial Insecticides among Populations of the Lesser Mealworm *Alphitobius diaperinus* Collected from Poultry Houses in France

**DOI:** 10.3390/insects12040309

**Published:** 2021-03-31

**Authors:** David Renault, Hervé Colinet

**Affiliations:** 1 Université de Rennes, CNRS, EcoBio (Ecosystèmes, Biodiversité, Évolution)—UMR 6553, 35000 Rennes, France; herve.colinet@univ-rennes1.fr; 2 Institut Universitaire de France, 1 rue Descartes, CEDEX 05, 75231 Paris, France

**Keywords:** pest beetle, poultry farms, pesticide resistance, toxicity, knockdown, mortality

## Abstract

**Simple Summary:**

The lesser mealworm *Alphitobius diaperinus* is a major pest from poultry houses worldwide. There was suspicion that populations of *A. diaperinus* had developed resistance to insecticides in poultry farms in France. Here, we evaluate the insecticide susceptibility of adult *A. diaperinus* from ten populations sampled from different poultry farms. The insects were exposed to four commercial insecticides: two pyrethroids, one pyrethroid/neonicotinoid and one organophosphate. Our results strongly suggest the occurrence of resistance to pyrethroid-based formulations in some farm populations from Brittany (France).

**Abstract:**

The control of insect pests often relies on the recurrent application of insecticides. This is the case for the lesser mealworm, *Alphitobius diaperinus*, an invasive beetle infesting poultry farms. There is evidence that *A. diaperinus* can develop resistance to several insecticides. Evaluation of such resistance has never been conducted in France, despite the beetle’s presence since the 1970s. We assess insecticide susceptibility in 10 populations from French poultry farms and compare patterns with two susceptible populations. Adults are subjected to short-term exposures (4 h) to four commercial insecticides and their recovery is assessed. Temporal survival also is scored during constant exposures for seven days. Clear-cut differences among the farm populations are found. Except for three populations that have patterns similar to those of the two susceptible populations, all the other farm populations have a much greater capacity to recover and survive insecticide exposures, especially to pyrethroid-based formulations. Three populations in particular even exhibit clear signs of resistance to pyrethroids, with median lethal times more than 10-fold superior to values of the susceptible population. No insect in any population recovers from the pirimiphos-methyl exposure, and all beetles are apparently dead after 15 h. Our results demonstrate the existence of resistant populations to pyrethroids in Brittany France.

## 1. Introduction

Many insect species are incurring significant costs and losses to agricultural production [1]. The control of insect pests has long relied on the intensive use of insecticide treatments [2], starting from a predominance of mineral treatments, and progressively moving to organic chemicals after the Second World War. Since then, the productivity issues posed by insect pests, and the lucrative adjacent market, have motivated the development and commercialization of chemicals having different modes of action [3]. The massive application of commercial chemicals has progressively favored the emergence of resistant populations [4], as evidenced in soybean aphids [5] or in the Colorado potato beetle [6]. The lesser mealworm *Alphitobius diaperinus* (Panzer) (Coleoptera: Tenebrionidae), a major pest from poultry houses worldwide, is not an exception (e.g., [7,8]).

The lesser mealworm is native to sub-Saharan regions [9], and was dispersed across the globe via the international foodstuff trade [10,11,12], particularly in stored products in the form of rice and cereals in which it thrives [7,13,14,15,16]. *A. diaperinus,* by infesting poultry farms, can be eaten by chickens [17], resulting in nutritional stress due to the limited capacity of birds to digest the chitin of insects [18]. *A. diaperinus,* by hosting a range of bacteria (*Escherichia coli, Salmonella enterica, S. typhimurium, Campylobacter jejuni*) [19,20,21,22] and viruses (for instance, infectious bursal disease, turkey coronavirus) [23,24], is also a vector of several diseases to poultry. Finally, this pest poses major problems to the structure of poultry buildings by digging large galleries into the insulation material when selecting favorable microhabitats for pupation [25]. Therefore, the development of large populations of *A. diaperinus* usually incurs significant economic losses (several million US dollars per year) to poultry production to control the outbreaks and repair the damage made to the buildings [26]. Even though new biological control strategies are developed [27,28,29,30,31,32], the use of chemical insecticides remains the main procedure for controlling populations of *A. diaperinus*. Depending on the nature of the poultry flock, e.g., chickens or turkeys, insecticides are sprayed every 7–14 weeks to disinfect the walls and litter at each building’s cleanout.

Since its first observation in 1977 in Brittany, France [10], several chemicals have been used in this region to control *A. diaperinus* population outbreaks, including organophosphates (tetrachlorvinphos), neonicotinoids (acetamiprid, imidacloprid) or pyrethroids (cyfluthrin, permethrin). While *A. diaperinus* populations develop in closed poultry houses, they are highly isolated from each other at the local and regional scales, i.e., the gene flow from outdoor populations is probably very limited and likely favors the selection of resistant individuals whose alleles can quickly propagate among the population. While recent works have demonstrated the efficiency of cypermethrin against adult *A. diaperinus* in Brazil and India [16,33], other investigations revealed that the susceptibility of larvae and adults to cypermethrin and chlorpyrifos can largely vary among populations from broiler facilities [15]. There also is increasing evidence that populations of *A. diaperinus* can develop resistance to insecticides, including bifenthrin, cyfluthrin, imidacloprid, permethrin, or tetrachlorvinphos [7,8,13,14,34,35,36]. 

Despite the fact that 40% of French poultry production is located in Brittany (France) [37], information on the susceptibility of *A. diaperinus* to insecticides in this region is lacking. Even more surprising is the absence of population resistance observations in France (http://www.pesticideresistance.org/ (accessed on 31 March 2021)), despite the significant pest nature of *A. diaperinus*. The assessment of the susceptibility of *A. diaperinus* to different commercial insecticides also is necessary to evaluate the existence of potential cross-resistance among insecticides [38,39]. This knowledge would provide guidance for improving integrated pest management strategies, such as the rotation of chemical products with different modes of action.

Here, we evaluate the insecticide susceptibility of adult *A. diaperinus* from ten populations sampled from different poultry farms in Brittany (France), and from two susceptible populations. The insects are exposed to four commercial insecticides: two pyrethroids, one pyrethroid/neonicotinoid and one organophosphate. The commercial formulations tested were, or are still, used for the disinfection of poultry buildings in France at the time of the study. We record the ability of insects to recover from a 4 h insecticide exposure, as well as the survival following a prolonged 7 d exposure. Large population outbreaks frequently occur in some poultry houses in Brittany despite regular insecticide applications [40]. We, therefore, expect to observe signs of resistance to commercial formulations in some populations, in particular for cyfluthrin, a pyrethroid that has been predominantly sprayed in the last decades in Brittany. We hypothesize that most resistant populations (Pop1–10) would be characterized by a faster recovery ability and higher survival compared to susceptible populations (PopS1 and S2). Finally, due to similar modes of action, we expect to find indications of cross-resistance between pyrethroid-based commercial products.

## 2. Materials and Methods

### 2.1. Collection of Insects

Insects were collected from ten poultry farms in November 2014 in various locations of Brittany (France) (Table 1). Adults of *Alphitobius diaperinus* were hand-collected from crevices and cracks, and from the litter nearby the feeders or along the walls of the buildings. Two additional susceptible populations (PopS1 and S2) also were used. PopS1, whose larvae are commercially distributed to serve as food for reptiles, was initially purchased from a pet shop (Envie Animales, Rennes, France). This industrial-reared population was most likely not treated with insecticides. PopS2 is a small population living in a cricket breeding facility at the Biological Station of Paimpont (University of Rennes, Rennes, France) in an insecticide-free environment for about 20 years.

Following collection, insects were maintained under controlled conditions (25 ± 1 °C, constant dark, and relative humidity ranging from 55 to 70%) in incubators (Thermostat cabinet TC 255 S, Lovibond). Each population was maintained isolated in a plastic box (27 × 28 × 8 cm, L × W × H) whose bottom was covered with a mix of sawdust and oat bran; the insects were supplied with dry dog food ad libitum, pieces of carrots, and Styrofoam™ to stimulate pupation. Water was supplied in pieces of cotton wetted with tap water. Mature adults were used for the experiments and newly emerged (reddish) adults were discarded. We did not differentiate the sexes for the experiments, and bioassays were conducted with adults only, as the insecticides used were adulticides.

### 2.2. Commercial Insecticides

We used four commercial insecticides (Table 2) commonly sprayed in poultry farms in Brittany. To simplify, the four commercial insecticides are further named using the active compound(s) they contain (Table 2).

### 2.3. Recovery Ability after Short-Term Contact Exposure

We assessed the recovery capacity of adults after short-term contact exposure to commercial insecticides. To that aim, a filter paper disc (diameter: 9 cm, surface: 63.6 cm^2^) was placed at the bottom of a plastic Petri dish (diameter: 9 cm; height: 2 cm). The four insecticides were dissolved in water, as indicated by the manufacturers, and the contact exposure was performed at the label rates corresponding to their recommended doses (RD) (Table 2). One mL of the insecticide was added to the filter paper of the Petri dish and left for 10 min to allow the complete absorption by the filter paper. Five Petri dishes, each containing 10 randomly collected adults of the same population, were labelled and prepared this way for each insecticide, and for each population (*n* = 5, *N* = 50). Regarding the control insects, only water (1 mL) was added to the filter paper. Occurring at the end of a 4 h contact exposure, adult *A. diaperinus* were transferred to insecticide-free Petri dishes, the bottom of which were covered with moistened filter paper (water only). The recovery of the adults was scored 4, 24, and 48 h after they were removed from the insecticide treatment, and the insects were categorized into a binary classification: (a) fit/recovered (i.e., walking adults with no jerky movements, visibly unaffected, as described by Lambkin [41]) or (b) weak/dead (i.e., erratic movements or lying on the back exhibiting only appendage movements or even no visible movement). The insecticide contact exposures and the subsequent recovery were performed at 25 ± 1 °C. The insects were food-deprived during the whole experiment.

### 2.4. Dynamics of Survival during Prolonged Contact Exposure

We examined the temporal survival profiles of the beetles continuously exposed to the same four insecticides for seven days. We used the same experimental design as the one described for short-term contact exposure, including the number of Petri dishes (replicates) and insects that were tested for each population and insecticide. To maintain humidity in the Petri dishes over the course of the experiment, the filter papers were watered (1 mL) every day. Insects were repeatedly scored for survival for seven consecutive days. Occurring at the start of the treatment (day 1), the survival of the insects was scored six times over the day, except for pirimiphos-methyl which required scoring for survival every 30 min because mortality occurred much faster for this insecticide than for the other molecules (i.e., all the insects were dead after only 24 h). Then, all insects were usually monitored two to four times a day. During each observation, the insects were categorized into a binary classification: (a) dead (i.e., no visible movement of any appendage even after mechanical stimulations) or (b) alive (i.e., walking and fit or moribund but still alive). The experiments were conducted at 25 ± 1 °C, and insects were deprived of food during the assays. Since the number of insects available was limited in the susceptible population PopS2, we used only the susceptible population PopS1 for this part of the study. Control insects were treated with water only and no mortality occurred during the 7 days of observation.

### 2.5. Statistical Analyses

Analyses and figures were performed using R 3.6.2 [42]. Regarding the differences in recovery ability according to the different populations (i.e., short-term contact bioassay), the data were analyzed using generalized linear models (GLMs) with a logit link function for binary outcomes (fit/recovered versus weak/dead beetles). The models were applied separately for the three different observation times (i.e., 4, 24, 48 h) and for the four different insecticides. A complete separation might have occurred in logistic/binomial regression when some categories contained 0 or 100% success (or failure); this was the case in some populations that showed 0 or 100% recovery at some stage. To account for that, a bias reduction method was implemented using “brglmFit” as the fitting method for GLMs within the “brglm2” R package [43]. The effects of the explanative variable (i.e., population) was then assessed via an analysis of deviance (“ANOVA” function in “car” R package; [44]).

To analyze and compare the patterns of survival/mortality according to the different populations and the time of exposure (i.e., prolonged contact bioassay), the data were analyzed using mixed effects generalized models (GLMMs) with a logit link function for binary outcomes (dead versus alive beetles). Since the insects within a replication (i.e., Petri dish) were monitored repeatedly during the time course of the experiment, the identity of replication (dish number) was used as a random variable to account for repeated measures. The effects of the two explanative variables (i.e., population and time) were analyzed via the analysis of deviance (“ANOVA” function in “car” R package; [44]). The differences among populations were analyzed by comparing least-squares means (Holm-adjusted) using the “emmeans” (Estimated Marginal Means) R package [45]. Additionally, for each population, the median lethal time (i.e., time to reach 50% mortality: LT50) was calculated as follows: LT50 = (logit (0.5) − *a*)/*b*, where *a* and *b* correspond respectively to the intercept and the slope of the GLM prediction [46]. Model parameters (LT50 and 95% CI) were then resampled (1000 iterations) thanks to the “arm” R package [47]. The LT50 values obtained also were confirmed and cross-validated using the function “dose.p” with 0.5 probability from the MASS R package.

## 3. Results

### 3.1. Recovery Ability

**Figure 1:**
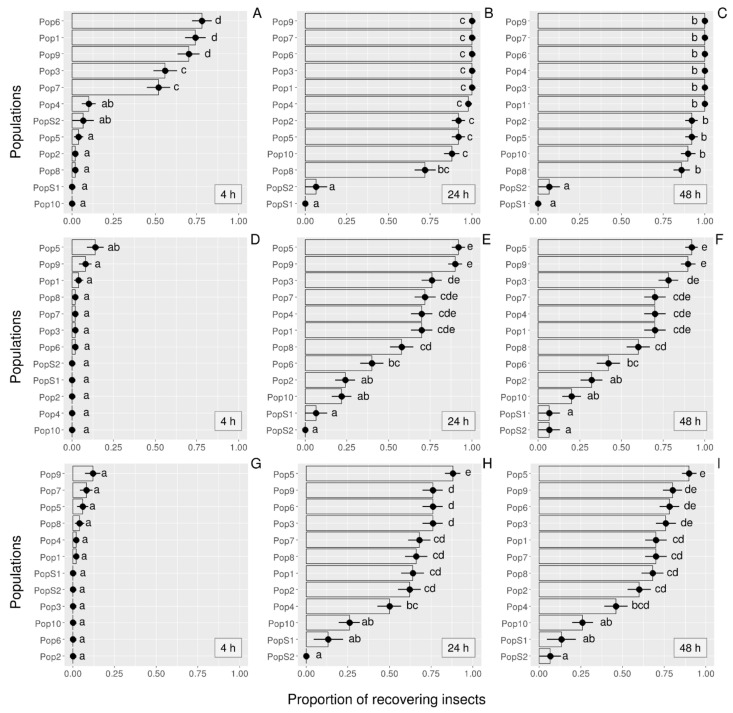
Mean proportions (±SE) of adult *Alphitobius diaperinus* that had recovered after a 4 h contact exposure to the recommended dose of cyfluthrin (**A**–**C**), permethrin (**D**–**F**), and acetamiprid + permethrin (**G**–**I**). Different letters indicate significant differences based on Holm-adjusted pairwise post hoc multiple comparisons. The recovery of the insects was scored 4, 24 and 48 h after they were transferred to an insecticide-free environment. See Table 1 and Table 2 for details on the origin of the twelve populations and the four commercial insecticides.

Regarding cyfluthrin, recovery ability markedly varied among populations (Figure 1A–C) at the three observation times (4 h: χ^2^ = 284.63, df = 11, *p* < 0.001; 24 h: χ^2^ = 190.84, df = 11, *p* < 0.001; 48 h: χ^2^ = 183.41, df = 11, *p* < 0.001). Following 4 h, the populations could be divided roughly into two groups: (i) those that mostly remained knocked down (i.e., Pop2, Pop4, Pop5, Pop8, Pop10, PopS1, PopS2) and (ii) those showing partial recovery, with about 50–75% of adults that had recovered (i.e., Pop1, Pop3, Pop6, Pop7, Pop9). Following 24 and 48 h, beetles from six farms had fully recovered, and recovery rates were about 85% in insects from the four other farms. Conversely, little or no recovery was observed in both susceptible populations (PopS1, PopS2).

The knockdown effect was particularly significant when the insects were treated with the pyrethroid permethrin (Figure 1D–F) or with the acetamiprid + permethrin formulation (Figure 1G–I). Concerning both treatments, recovery was quasi null after 4 h in all tested populations. The proportion of beetles that had recovered significantly differed among populations and increased in most of the populations from poultry farms after 24 h (permethrin: χ^2^ = 172.07, df = 11, *p* < 0.001; acetamiprid + permethrin: χ^2^ = 104.46, df = 11, *p* < 0.001) and 48 h of recovery (permethrin: χ^2^ = 157.03, df = 11, *p* < 0.001; acetamiprid + permethrin χ^2^ = 109.09, df = 11, *p* < 0.001). None of the farm population had fully recovered 48 h after the exposure; the maximum recovery rate was measured in adults from Pop5 and Pop9 (75–90%) for both molecules. Pop2 and Pop10 showed relatively poor recovery ability after exposure to permethrin. Again, recovery was very low in both susceptible populations (PopS1 and PopS2) after a short exposure to permethrin or acetamiprid + permethrin.

Four, 24 and 48 h post-treatment, no insect in any population had recovered from the pirimiphos-methyl exposure. Using this insecticide, after the treatment, most insects remained weak or eventually died after 24 h (i.e., lying on their backs with only movement of their appendages or no visible movement at all).

### 3.2. Survival Rates

**Figure 2:**
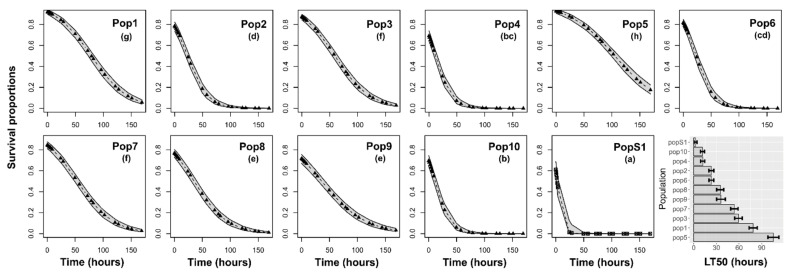
Survival probability of adults according to the time of exposure to cyfluthrin. Insects were exposed for seven days to the recommended dose and scored for mortality at multiple time points during the exposure. The estimated median lethal times (LT50) are presented for each population in the histograms at the right bottom of the panel (with 95% confidence intervals). Different letters in brackets above the curves indicate differences among populations based on Holm-adjusted pairwise post hoc multiple comparisons.

**Figure 3:**
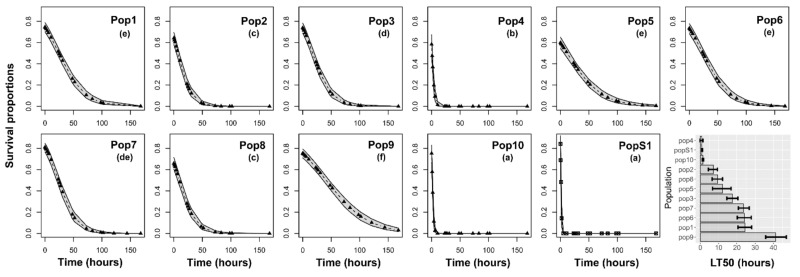
Survival probability of adults according to the time of exposure to permethrin. Insects were exposed for seven days to the recommended dose and scored for mortality at multiple time points during the exposure. The estimated median lethal times (LT50) are presented for each population in the histograms at the right bottom of the panel (with 95% confidence intervals). Different letters in brackets above the curves indicate differences among populations based on Holm-adjusted pairwise post hoc multiple comparisons.

**Figure 4:**
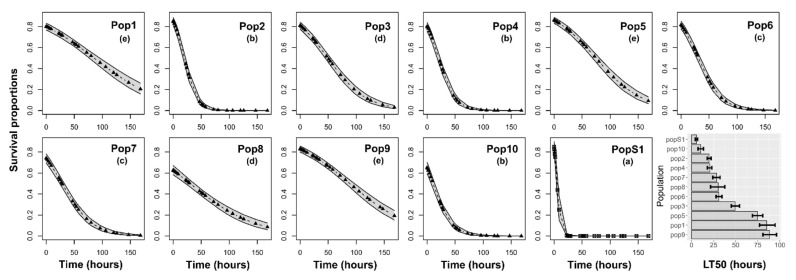
Survival probability of adults according to the time of exposure to acetamiprid + permethrin. Insects were exposed for seven days to the recommended dose and scored for mortality at multiple time points during the exposure. The estimated median lethal times (LT50) are presented for each population in the histograms at the right bottom of the panel (with 95% confidence intervals). Different letters in brackets above the curves indicate differences among populations based on Holm-adjusted pairwise post hoc multiple comparisons.

**Figure 5:**
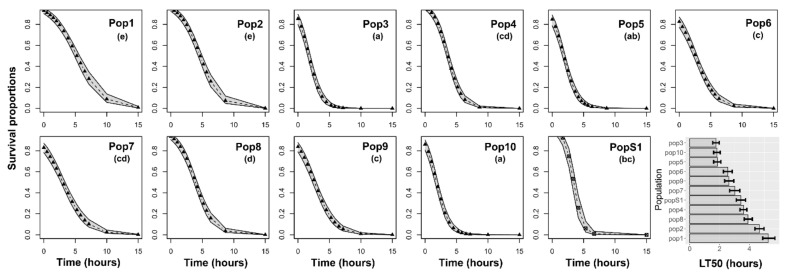
Survival probability of adults according to the time of exposure to pirimiphos-methyl. Insects were exposed for seven days to the recommended dose and scored for mortality at multiple time points during the exposure. The estimated median lethal times (LT50) are presented for each population in the histograms at the right bottom of the panel (with 95% confidence intervals). Different letters in brackets above the curves indicate differences among populations based on Holm-adjusted pairwise post hoc multiple comparisons.

Large survival differences among populations were found for insects subjected to cyfluthrin (χ^2^ = 221.90, df = 10, *p* < 0.001) (Figure 2), permethrin (χ^2^ = 70.40, df = 10, *p* < 0.001) (Figure 3), acetamiprid + permethrin (χ^2^ = 145.13, df = 10, *p* < 0.001) (Figure 4), and pirimiphos-methyl (χ^2^ = 158.16, df = 10, *p* < 0.001) (Figure 5). Regarding the four insecticides, time of exposure significantly reduced the survival rate (cyfluthrin: χ^2^ = 299.02, df = 1, *p* < 0.001; permethrin: χ^2^ = 162.20, df = 1, *p* < 0.001; acetamiprid + permethrin: χ^2^ = 137.72, df = 1, *p* < 0.001; pirimiphos-methyl: χ^2^ = 158.16, df = 1, *p* < 0.001). The temporal patterns also varied according to the populations, resulting in a significant time x population interaction (cyfluthrin: χ^2^ = 202.24, df = 10, *p* < 0.001; permethrin: χ^2^ = 245.56, df = 10, *p* < 0.001; acetamiprid + permethrin: χ^2^ = 460.14, df = 10, *p* < 0.001; pirimiphos-methyl: χ^2^ = 108.29, df = 10, *p* < 0.001). All estimated median lethal times (i.e., LT50 values) along with their 95% confidence intervals are given in Supplementary Table S1 together with the fold change (FC) variations relative to the LT50s of the susceptible population.

Regarding cyfluthrin (Figure 2), PopS1 exhibited the fastest mortality, with an LT50 value of only 2.35 h. Insects from Pop4 and Pop10 also were highly affected by cyfluthrin treatment (LT50 of 11.8 and 11.4 h, respectively). Pop1 and Pop5 were relatively good at surviving cyfluthrin formulation, with LT50 values of 78.4 and 105.1 h, respectively (Supplementary Table S1).

Regarding permethrin (Figure 3), the lowest survival rates were recorded in PopS1, Pop4, and Pop10 (LT50 of 0.97, 0.75, and 1.39 h, respectively) and the highest survival was found in adults of Pop9 and Pop5 (LT50 of 41.13 and 24.54 h, respectively) (Supplementary Table S1).

Regarding acetamiprid + permethrin (Figure 4), the lowest survival rates were recorded for PopS1, Pop10, Pop2, and Pop4 with LT50 values of 5.65, 10.52, 19.9, and 20.68 h, respectively. The highest survival rates were measured in Pop5, Pop1, and Pop9, with LT50s of 74.78, 85.70, and 88.56 h, respectively (Supplementary Table S1). Concerning these three latter populations, some insects still were alive at the end of the experiment, i.e., after 7 d of constant exposure to the insecticide.

The pirimiphos-methyl formulation (Figure 5) resulted in the death of almost all insects within only 24 h post-exposure. Pop3, Pop5, and Pop10 were the most susceptible with an estimated LT50 of about 1.76, 1.86, and 1.82 h, respectively. PopS1 was intermediated (LT50 of 3.42 h) and Pop1 survived the longest with LT50 values of only 5.27 h (Supplementary Table S1).

## 4. Discussion

Our results, for the first time to our knowledge, evidence the loss of pyrethroids’ efficiency against farm-collected *Alphitobius diaperinus* in France. Indeed, most populations from poultry farms were less susceptible to pyrethroid-based formulations compared to susceptible populations that had been maintained in a pesticide-free environment for many generations. Except for Pop2, Pop4, and Pop10, which were characterized by survival patterns roughly similar to that of the susceptible populations, all other populations had a much greater capacity to recover and survive insecticide exposure. Pop1, Pop5, and Pop9 exhibited strong signals of resistance to pyrethroid-based formulations, with median lethal time (LT50) values being consistently more than 10-fold superior to that of the susceptible population (PopS1 and S2) (Supplementary Table S1). Regarding pyrethroids, LT50 values ranged from a few hours to several days, resulting in differences relative to the susceptible population reaching up to 44-fold for cyfluthrin and 42-fold for permethrin. This large population-based variability likely implies that the different histories of exposure to insecticides have contributed to a distinct evolution of insecticide resistance among populations. This assumption is in line with the work of Singh and Johnson [38] who compared the susceptibility to cyfluthrin and tetrachlorvinphos of *A. diaperinus* sampled from two chicken broiler farms. These authors reported that lesser mealworms from the farm where disinfection procedures were systematic for 10 years before the commencement of each flock were generally much more resistant to both chemicals. Conversely, insects from the farm which had no insecticide usage history had maintained a high susceptibility to cyfluthrin and tetrachlorvinphos [38].

The recovery tests revealed that all farm populations efficiently and uniformly recovered from cyfluthrin exposure. Even after 4 h of recovery, half of the tested lesser mealworms had already recovered, which suggests an ineffectiveness of this molecule against the tested farm populations. This observation is consistent with the available knowledge on the susceptibility of *A. diaperinus* to cyfluthrin [38,39]. All told, 28 populations of *A. diaperinus* tested in the time range 2011–2015 in Australia and the U.S.A. were listed as resistant to cyfluthrin [38,39]. Likewise, Hamm et al. [7], who worked with the pure cyfluthrin molecule at the LC95, obtained resistance ratios ranging from 1.7 to 9.5-fold for adults, and ranging from 0.5- to 29-fold for larvae.

The extensive and repeated usage of insecticides is well known to favor the development of resistance [48]. Several studies have reported moderate to high resistance levels to a range of insecticides (carbaryl, chlorpyrifos, cyfluthrin, cyhalothrin, cypermethrin, deltamethrin, fenitrothion, methoxychlor, tetrachlorvinphos) in *A. diaperinus* sampled from poultry houses [8,38,39,49,50]. Here, resistance to pyrethroids was particularly striking in Pop1, Pop5, and Pop9 for which constant exposure for seven consecutive days was not long enough to kill 100% of the insects. Pyrethroid resistance can rapidly develop in populations of *A. diaperinus* and a direct correlation between the number of cyfluthrin applications and its resistance has been evidenced by Lambkin and Rice [49]. Specifically, these authors found that cyfluthrin resistance can be 22-fold higher in lesser mealworms from farms that have systematically been sprayed with the insecticide at the commencement of the flock over the past four years (corresponding to 20 insecticide disinfections in total) compared to susceptible populations. Regarding Brittany, cyfluthrin formulation, with the patent granted to SOLFAC^®^ 10 dating back to 1995, has been massively sprayed in poultry houses. The reduced efficiency of cyfluthrin in populations from Brittany (France), thus, is not so surprising and consistent with former reports of resistance to cyfluthrin in Australia and the USA [38,39].

Similarly, permethrin formulation was characterized by a low efficiency against poultry farm-collected populations of *A. diaperinus*. However, most insects were knocked down just after the exposure, as opposed to the results obtained by Lyons et al. [36] who suggested a limited knockdown effect of an exposure for 2 h by adult *A. diaperinus* to permethrin. During our study, more than half of the insects had recovered 48 h after the exposure, which was not the case for susceptible populations. Low mortality was recorded in the six populations exposed to permethrin during the bioassays performed by Tomberlin et al. [51] who used an exposure time of only 1 h. While we obtained similar results after 1 h of exposure, survival was close to zero in most populations after several days of exposure to permethrin. The marked temporal variations we observed emphasize the need for longitudinal observations over at least 24–48 h when assessing the toxicological effects of insecticides. Regarding cyfluthrin, the temporal dynamics of mortality was much faster in the susceptible population; LT50 values were more than 10-fold higher than in the susceptible insects for at least half of the farm populations, suggesting that a permethrin-based formulation is inefficient for controlling some of the lesser mealworm populations in Brittany.

The formulation combining both permethrin and acetamiprid resulted in a high recovery and relatively high LT50s in several poultry farm-collected populations, while susceptible populations did not recover and quickly died. Overall, this product had a slightly higher efficiency (relative to susceptible insects) than cyfluthrin or permethrin alone. Only three farm populations (Pop1, Pop5, Pop9) had LT50 values 10-fold higher than the susceptible population. This finding might be related to the addition of 3% acetamiprid in the formulation, a chemical having a high toxicity to wildlife in general, and whose use is prohibited in France since 2018. Yet, the acetamiprid formulation we tested still was marketed at the collection time of the insects (it received a marketed authorization in the middle of the year 2013, [52]). The adding of other chemicals to pyrethroids often has been suggested to fully, or partially, restore the efficiency of chemical insecticides, as demonstrated with spinosad and cyfluthrin, whose synergistic interaction improves the control of cyfluthrin-resistant populations of the lesser mealworm [39]. The limited duration of the commercialization of acetamiprid + permethrin, together with the high toxicity of the two chemicals in mixture, are consistent with the slightly higher efficiency we noted relative to the two other pyrethroid-based formulations.

To contrast to the three other insecticides discussed above, the formulation based on pirimiphos-methyl was still highly efficient against *A. diaperinus*, resulting in a very fast mortality in all tested populations, and LT50 values close to that of the susceptible population. This result is consistent with the current absence of resistance reported for pirimiphos-methyl in the lesser mealworm (http://www.pesticideresistance.org/ (accessed on 31 March 2021)). Importantly, this insecticide has been authorized in France since late 2012 only (https://ephy.anses.fr/ppp/pirigrain-250 (accessed on 31 March 2021)), suggesting that its application in poultry houses may have been quite limited. The high toxicity of pirimiphos-methyl has been reported for several other insects, including the rice weevil *Sitophilus oryzae* (Linneaus) (Coleoptera, Curculionidae) [53], the wheat weevil *Sitophilus granaries* (Linneaus) (Coleoptera, Curculionidae), and the khapra beetle *Trogoderma granarium* (Everts) (Coleoptera, Dermestidae) [54]. It also causes high mortality to small larvae of the tenebrionid *Tenebrio molitor* (Linneaus) (Coleoptera, Tenebrionidae) [55]. Additionally, it has been demonstrated that this chemical can have long-lasting effects on the red flour beetle *Tribolium castaneum* (Herbst) (Coleoptera, Tenebrionidae), with parental exposure affecting the fitness of the progeny, in particular when contact exposure duration was increased [56]. The high toxicity of pirimiphos-methyl toward insects in general may explain its absence from the list of organophosphates that can be sprayed for controlling *A. diaperinus* populations in poultry houses in Arkansas (U.S.A.) [57]. However, there are populations from 20 arthropod species for which resistance to pirimiphos-methyl also has been reported (http://www.pesticideresistance.org/ (accessed on 31 March 2021)), including in the tenebrionid *T. castaneum* [53], hence strategies for avoiding resistance also should be considered when using this molecule.

Collectively, our results (both recovery and mortality assays) report the high efficiency of pirimiphos-methyl for controlling *A. diaperinus* outbreaks in poultry farms, and strongly suggest the occurrence of resistance to pyrethroid-based formulations in some farm populations (e.g., Pop1, Pop5, Pop9). The data also showed that other populations (e.g., Pop2, Pop4, Pop10) retained a high susceptibly to pyrethroids, similar to that of the two susceptible populations. The former exposure histories to the chemicals are probably responsible for the variation in sensitivity among the different populations, as also reported by Chernaki-Leffer et al. [58]. Despite some variation in the responses among the three pyrethroid pesticides tested, we found that, overall, Pop1, Pop5, and Pop9 were consistently among those that recovered most effectively and survived pesticide exposure the longest. This suggests the possible existence of a cross-resistance phenomenon to pyrethroid-based formulations that share the same mode of action. Cross-resistance among tetrachlorvinphos and cyfluthrin in *A. diaperinus* also was suggested by Hamm et al. [7]. To parallel, we also report that susceptible populations still can be found in Brittany (France). It must be noted that we did not feed insects during the assays, so we cannot rule out that food deprivation might have slightly overestimated mortality. Yet, this must be rather limited as food-deprived adult *A. diaperinus* can survive up to 10 days without any significant mortality [59]. We suggest as an opening perspective, that further investigations should be conducted to assess the susceptibility to pure compounds and the mechanisms underlying insecticide resistance in the lesser mealworm. It would be helpful to gain knowledge on the possible existence of behavioral avoidance and metabolic resistance in resistant populations. Finally, our study re-emphasizes the crucial need to modify and alternate the insecticides used among the different flocks. The rotation of chemicals with distinct modes of action is essential, as a part of integrated management plans of poultry house facilities. The efficiency of this procedure, and its ability to prevent insecticide resistance development, also greatly depends on the regular testing of the susceptibility of the pest populations.

## Figures and Tables

**Table 1:** Presentation of the twelve populations of *Alphitobius diaperinus* whose susceptibility to commercial insecticides was assayed. Populations 1 to 10 (coded as Pop1–Pop10) were sampled from poultry farms. The susceptible populations (coded as PopS1 and PopS2) originated from insecticide-free rearing and laboratory cultures over many generations.

Origin of the Population	Geographic Coordinates (WGS84)	Designation
Allaire	47°38′13″ N, 02°09′46″ W	Pop1
Crevin	47°56′14″ N, 01°39′42″ W	Pop2
Guéhenno	47°53′32″ N, 02°38′22″ W	Pop3
Guer	47°54′14″ N, −2°07′14″ W	Pop4
Limerzel	47°38′12″ N, 02°21′14″ W	Pop5
Plumelec	47°50′16″ N, 02°38′27″ W	Pop6
Ruffiac building 1	44°21′43″ N, 00°01′60″ E	Pop7
Ruffiac building 2	44°21′43″ N, 00°01′60″ E	Pop8
St Martin sur Oust	47°44′46″ N, 02°15′15″ W	Pop9
Taupont	47°57′31″ N, 02°26′19″ W	Pop10
Sensitive1 (Rennes)	48°06′51″ N, 01°40′51″ W	PopS1
Sensitive2 (Paimpont)	48°01′11″ N, 02°15′2″ W	PopS2

**Table 2:** Description of the four commercial insecticides. The brand name, the dosage, and the chemical family of the active compound(s), the mode of action of the active molecule, and the label rates (doses recommended by the manufacturer) are presented.

Brand Names	Active Compound and Dosage	Chemical Family	Mode of Action	Recommended Dose (RD) Per Surface Unit
SOLFAC 10	Cyfluthrin 10%	Pyrethroid	Inhibits the closure of voltage-gated sodium channel	50 mL/m^2^
TOP KILL 10	Permethrin 10%	Pyrethroid	Inhibits the closure of voltage-gated sodium channel	2 mL/m^2^
LEXAN 30/PER 120 (SECTIN)	Acetamiprid (30 g/L)	Neonicotinoid	Binds to nicotinic acetylcholine receptor	1.6 mL/m^2^
	Permethrin (120 g/L)	Pyrethroid	Inhibits the closure of voltage-gated sodium channel	
PIRIGRAIN 250	Pirimiphos-methyl 24.04%	Organophosphate	Inhibits acetylcholinesterase	0.8 mL/m^2^

## Data Availability

Data are contained in the article.

## Supplementary Materials

The following are available online at https://www.mdpi.com/article/10.3390/insects12040309/s1, Table S1: Estimated median lethal times (LT50 values) and their upper/lower 95% confidence intervals (CI) for the different populations exposed to 4 different insecticides for 7 consecutive days.
